# Severe Bicuspid Aortic Valve Disease in an Adult Cystic Fibrosis Patient

**DOI:** 10.7759/cureus.50401

**Published:** 2023-12-12

**Authors:** Jason Y Zheng, Samra Haroon Lodhi, Michael Anstead, Naoki Misumida, Taha Ahmed

**Affiliations:** 1 Medicine, University of Kentucky College of Medicine, Lexington, USA; 2 Internal Medicine, University of Kentucky College of Medicine, Lexington, USA; 3 Pulmonology, University of Kentucky College of Medicine, Lexington, USA; 4 Cardiology, University of Kentucky College of Medicine, Lexington, USA

**Keywords:** cystic fibrosis, surgical aortic valve replacement, tavr (transcatheter aortic valve replacement), aortic valve stenosis, bicuspid aortic valve

## Abstract

Bicuspid aortic valve is the most common congenital heart disease. Bicuspid aortic valves are prone to accelerated degenerative changes and aortopathies. These changes often manifest in adulthood as severe aortic stenosis or mixed aortic valve disease. Cystic fibrosis patients are at high risk of adverse surgical outcomes. As survival in cystic fibrosis continues to increase, managing comorbidities including severe aortic stenosis requires consideration. The relatively non-invasive transcatheter aortic valve replacement has been posed as an intervention for high-risk patients with severe symptomatic aortic stenosis. However, traditional randomized trials have excluded patients with bicuspid aortic valves. Herein we present an extremely rare association of severe bicuspid aortic valve stenosis in an adult cystic fibrosis patient. Furthermore, we discuss the clinical course and a multi-disciplinary approach for the management of this rare scenario.

## Introduction

Aortic stenosis (AS) is a commonly encountered valvular heart disease that is characterized by a narrowing of the aortic valve. This is most often due to age-related calcifications that impede the blood flow across the stenotic valve. Congenital malformations to the aortic valve such as bicuspid aortic valve (BAV) predispose younger patients to this wear-and-tear disease [1]. This outflow obstruction leads to the typical symptoms of angina, exertional dyspnea, and syncope. Traditionally, AS was treated by surgical aortic valve replacement (SAVR), but within the last two decades, the less invasive transcatheter aortic valve replacement (TAVR) has gained favor. Randomized data looking at outcomes between the two methods largely excludes the population of patients with BAV, which has limited the role of TAVR in bicuspid AS [1]. This paucity of data is significant because BAV disease accelerates the rate of valvular calcification, leading to an earlier presentation of symptomatic AS [1]. Management may further be complicated by concomitant severe chronic lung disease, which if co-existent with severe AS, serves as a deterring factor for the traditional SAVR approach [2]. Cystic fibrosis (CF) is an autosomal recessive multisystem disease that occurs due to mutations in the CF transmembrane conductance regulator gene [3]. There is a scarcity of existing data that investigates the association between the two pathologies. Here in this article, we present a case of severe aortic valve disease complicated by underlying CF and bicuspid valve abnormality. 

## Case presentation

A 28-year-old man with a past medical history of CF and congenital AS, previously at baseline state of health, presented to the emergency department due to a chief complaint of hemoptysis. The patient noticed expectoration of frank blood and described a feeling of blood filling his lungs with respirations. He reported a blood volume expectorated estimated to be around 60 mL. The patient denied chest pain, dyspnea, or fevers. The patient reported that he had a previous episode of hemoptysis after being on lumacaftor/Ivacaftor for two and a half months. 

The patient’s past surgical history consisted of surgical aortic valvulotomy during childhood due to congenital AS. The patient had no family history of CF; however, an unknown cardiac disorder in his maternal grandmother was reported. The patient denied any tobacco, alcohol, or recreational drug use. On presentation, vital signs were stable with a blood pressure of 127/84 mmHg, temperature of 98.5 F, heart rate of 99 beats per minute, and a respiratory rate of 18 per minute on room air. On exam, the patient was well-appearing with no acute distress. Cardiac rate and rhythm were regular with a grade IV/VI early peaking systolic ejection murmur auscultated over the precordium. Crackles were auscultated in the left upper fields and expiratory wheezes were in the left lower lung fields. Of note, his latest forced expiratory volume in one second (FEV1) was 40% predicted at his pulmonology office visit two weeks prior. Laboratory findings were significant for leukocytosis with WBC 15x10^9^/L (normal: 3.7-10.3) and an albumin level of 2.6g/dL (normal: 3.3-4.6).

Chest X-ray revealed an area of focal consolidation. CT angiography demonstrated a hypertrophied bronchial artery extending into the left hilum and bronchiectasis-related plugging in the left upper lobe. Systemic tobramycin and ceftazidime for pseudomonas coverage were initiated. However, the bleeding worsened during the hospital stay necessitating intubation and bronchoscopic evaluation, which revealed a visible clot in the left upper lobe and purulent secretions in the right upper lobe. Interventional radiology-guided embolization of the left bronchial artery and left bronchial branch of the right bronchial artery was pursued with effective cessation of the bleeding. Treatment was successful and the patient was discharged with diagnoses of hemoptysis and CF exacerbation. For further workup of the precordial murmur, outpatient transthoracic echocardiography and cardiology follow-up were suggested. 

Outpatient transthoracic echocardiogram (TTE) after discharge illustrated a BAV with thickened leaflets and severe AS (mean and maximum pressure gradients were 50 and 86 mmHg, respectively) with mild regurgitation. Left ventricular ejection fraction (LVEF) was 55%. The patient was seen at our adult congenital heart disease clinic, and a cardiac MRI was planned. 

**Figure 1:**
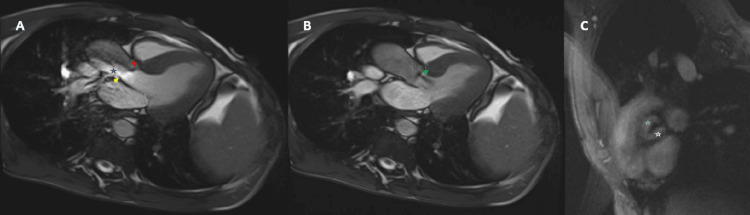
Cardiac MRI Cardiac MRI showing A) three-chamber view in long axis during systole showing severe bicuspid aortic stenosis with a large anterior leaflet (red arrow), smaller posterior leaflet (yellow arrow), and eccentric jet of blood (blue star) across the valve B) during diastole showing mild aortic regurgitation jet (green arrow) C) short-axis view showing bicuspid aortic valve with large anterior (green star) and small posterior leaflet (white star).

Four months after the index hospitalization, the patient had another hospitalization for similar issues of dyspnea, hemoptysis, and CF exacerbations. Conservative management with antibiotics was performed during this hospitalization. At 11 months after the index hospitalization, the patient had another hospital admission for severe shortness of breath, increased sputum production, and hemoptysis requiring transfer to the ICU. A repeat TTE showed an aortic valve area of 0.9 cm^2^ and a mean gradient of 40 mm Hg across the aortic valve. It was deemed that there was no urgent need for a surgical or transcatheter aortic valve replacement. Subsequently, an outpatient cardiac MRI was obtained, which demonstrated a BAV with severe stenosis and mild regurgitation (Figure 1A-1C).

Peak velocity was > 5 m/s. The ascending aortic was mildly dilated with the maximum being 39 mm, the left ventricle had normal size, and the ejection fraction was 62%. 

A heart team discussion was undertaken. Overall, aortic valve replacement via either surgical or transcatheter aortic valve replacement was deferred largely due to the lack of symptoms of exertional syncope, dizziness, and lightheadedness. Complications associated with the patient’s CF-related lung disease and malnutrition were additional aspects to consider. 

On outpatient follow-up, the most recent transthoracic echocardiography demonstrated LVEF between 55 and 60%. Aortic valve cusp calcifications were noted. Mean and maximum gradient pressures were 44 and 70 mmHg, respectively. The dimensionless index was noted to be 0.25-0.5. The latest pro-BNP was measured to be within normal limits at 171 pg/mL (normal: 0-449). Of note, the patient had frequently been anemic during his course of disease. Hemoglobin measurements had mostly ranged from 9 to 13 g/dL. The last FEV1 measurement was 45% predicted. Currently, the benefits of undergoing procedural management are limited due to the absence of symptoms related to severe AS.

## Discussion

There is limited existing literature that investigates the association between CF and coexisting AS. This is partially due to the polarizing age groups that these diseases primarily affect. CF being a genetic condition often leads to mortality early in one’s life, secondary to end-stage lung disease, which is the most common etiology of death [3]. AS as a wear-and-tear disease typically manifests in the elderly population. Valvular calcification was noted in 75% of individuals in the 80s in the Helsinki Aging Study [4]. This discrepancy is one of the reasons for the paucity of data in the literature. However, with the development of better pharmacological therapy, the life expectancy of CF patients has steadily increased over the recent years. Currently, over 50% of patients with CF are adults and median survival in the United States has risen to 56 years according to the CF Foundation Patient Registry 2022 Annual Data Report [5].

Currently, some research supports an association between CF and structural impairment of elastic properties of the aorta [6]. In the small case-control study, investigators found a statistically significant decrease in distensibility and an increase in stiffness of the aorta in CF patients when compared to healthy controls [6]. Furthermore, in murine models, there has been evidence of increased aortic stiffness in mice with CF-related mutations [7]. Additional research looking at the management of severe AS with concomitant CF may be most impactful in patients with a congenital BAV. This is due to the earlier onset of symptomatic AS associated with a bicuspid valve. Currently, management guidelines do not exist for this specific patient population, which would greatly benefit the patient in our case report. The need for additional research continues to increase as the lifespans for our CF patients continue to climb. 

One of the key features in this patient’s case is the root cause of the shortness of breath. The dyspnea could either be due to CF or severe AS. This is important in the clinical management of this patient’s valvular disease due to the timing of intervention. Typically, AS is acted upon when the patient becomes symptomatic with complaints of syncope, angina, and exertional dyspnea. Management typically takes the form of aortic valve replacement either through the surgical or transcatheter approach. 

Asymptomatic AS is associated with low rates of sudden death and thus is not treated with valve replacement if without other indications [4]. Even in patients with severe asymptomatic AS with peak velocity > 4 m/s, the chances of remaining free of cardiac symptoms at one, two, and five years are 82%, 67%, and 33%, respectively [4]. This is the case for our patient, who remains relatively asymptomatic (without symptoms of angina or syncope) and has maintained an LVEF of approximately 55-60%. This explains most of the hesitation to provide any sort of intervention, whether that be via SAVR or TAVR. On the other hand, the patient’s FEV1 has shown marginal improvement from 40% to most recently 45% predicted over these years of hospitalizations. It is likely that there exists some interplay between the two different etiologies. It is important to tease out the predominant cause in order to better customize therapeutic strategies for the most beneficial outcomes. 

AS has been reported as the most common cause of valve replacement in patients with BAV in 61% to 88% of population-based studies and those from tertiary referral centers [1]. Data supporting the best method of intervention is lacking for these patients because bicuspid valve cases are largely excluded from the historical randomized studies that compared the SAVR vs. TAVR approach. 

TAVR is the newer approach through a less invasive mechanism that has gained popularity in the treatment of tricuspid AS in the last decade. A study that examined the temporal trends of aortic valve replacement strategies in the American population revealed that the usage of TAVR has risen to a comparable frequency in patients with BAV in 2016 [8]. The study also revealed no statistical significance in in-hospital mortality between the two treatments. Hemodynamic performance in TAVR is superior to that of SAVR [9]. There is a decrease associated with acute myocardial infarction and the need for blood transfusions when compared to patients undergoing SAVR, obviously due to the less invasive nature of the procedure. The most common complication of TAVR is the need for permanent pacemaker implantation (PPI). No difference in mortality has been observed in patients who required a PPI after SAVR or TAVR, but the procedure still increases the duration of hospitalization and rate of rehospitalization [9].

Looking at the SAVR approach, the major indication is in low-risk patients with severe multi-vessel coronary artery disease [4]. One of the major benefits of performing SAVR in patients with bicuspid aortic disease is to simultaneously perform additional procedures that may be indicated. Patients with BAV often concomitantly have some sort of aortopathy, with aortic dilation presenting in up to 40% of cases [1]. The patient presented in this case had ascending aorta dilation with a maximum diameter of 39 mm noted on cardiac MRI. This poses a problem for TAVR because the replacement valve is not fixated in place manually but rather relies on the surrounding anatomy to secure the positioning. A dilated aortic root would present difficulties with preventing the replacement valve from slipping. Additionally, SAVR is preferred in cases that may require reintervention in the future. This is important for our patients with BAV since they often present earlier for intervention. In patients with BAV disease that undergo aortic valve replacement for severe AS, 60% are younger than 70 years old [4]. SAVR is also preferred in cases with concomitant valvulopathies or coronary heart disease that can be operated upon at the same time [1]. The largest limitation of SAVR is in patients with hesitations to undergoing surgical intervention. Lung disease is a risk factor for morbidity and mortality after SAVR [2]. Another point of discussion when considering SAVR is whether to implement a mechanical or bioprosthetic heart valve. Given this patient’s young age, a mechanical valve would be preferred given the benefit of increased longevity of the valve. However, mechanical valves require anticoagulation therapy, which may be problematic in our patient with frequent episodes of hemoptysis due to his underlying CF further complicating the decision-making. 

To the author’s knowledge, this is one of the first reported cases of severe AS in a CF patient and highlights the complexity of management. While traditional TAVR and SAVR risk factors do incorporate lung disease, the concerns in CF patients also involve frequent infections with resistant bacterial organisms. Although CF patients have chronic lung infection, bacteremia in CF is relatively infrequent [10].

The ultimate decision for our patient was to not provide intervention at the time based on the lack of symptoms. TAVR and SAVR have similar incidences of developing postop infectious endocarditis at about 0.3 to 2.0 per 100 person-years [11]. This is a significant complication that is associated with high rates of in-hospital mortality. There is no information in the literature to determine if CF patients undergoing TAVR or SAVR have a risk for postoperative infectious endocarditis that is meaningfully increased. Frequent hospitalizations and antibiotic usage do predispose CF patients to infections by antibiotic-resistant microbes. This could make effective treatment with antimicrobials more challenging if the patient with CF did develop postop infectious endocarditis. 

CF patients may experience additional risk factors for TAVR. TAVR is effective in the treatment of AS in the general population but is associated with a lack of clinical improvement in certain subgroups. There is some evidence that patients with oxygen-dependent chronic lung disease, poor baseline mobility, and low BMI experience futility with TAVR [2]. Our patient has a BMI of 21 kg/m^2^, which is within normal limits; however, patients with CF with severe lung disease are at a higher risk for malnutrition. Speaking more specifically on the prognosis of chronic lung disease patients undergoing TAVR, numerous national registries have revealed earlier post-op mortality when compared to matched controls without lung pathology [12]. 

Patients with a congenital BAV are at increased risk of AS. Large amounts of data exist supporting the usage of TAVR or SAVR in the treatment of AS in tricuspid valves. However, there is a paucity of literature guiding the management of AS in BAV. Severe AS can be associated with concomitant chronic lung disease, which further complicates treatment in these populations. The role of aortic valve replacement and the interplay between AS and chronic lung disease is important in patients with bicuspid valves due to the predisposition to requiring intervention. In this case report, we look at the considerations for management in a patient with CF with concomitant severe AS. With the improved life expectancy in CF, it is likely that these conditions will co-exist more frequently. 

Further studies are needed to establish ideal management pathways for this cohort; as of now, management with either a TAVR or SAVR cannot be commented upon due to a lack of evidence in CF patients. Nonetheless, the decision-making regarding the treatment of these patients is multifactorial and should be approached with a multi-disciplinary approach that incorporates patient-specific factors and goals. 

## Conclusions

With the improvement in CF treatment and the subsequent increase in life expectancy, diseases of aging such as AS are becoming more relevant in this patient population. In a patient with CF-related lung disease and AS, it can be difficult to decipher whether dyspnea is secondary to pulmonary or cardiac etiology, but it is a critical determination that affects therapeutic management. Decision-making for the repair of bicuspid AS in patients with CF-related chronic lung disease is a complex discussion that involves multiple factors and should incorporate patient goals. SAVR and TAVR are both options that have different associated benefits and difficulties; however, no evidence validates the utility of either modality in CF patients. 
